# Systemic administration of quality- and quantity-controlled PBMNCs reduces bisphosphonate-related osteonecrosis of jaw-like lesions in mice

**DOI:** 10.1186/s13287-019-1308-8

**Published:** 2019-07-16

**Authors:** Shinichiro Kuroshima, Kazunori Nakajima, Muneteru Sasaki, Takashi I, Yoshinori Sumita, Takayuki Asahara, Izumi Asahina, Takashi Sawase

**Affiliations:** 1 Department of Applied Prosthodontics, Institute of Biomedical Sciences, Nagasaki University, 1-7-1, Sakamoto, Nagasaki, 852-8588 Japan; 2 Department of Regenerative Oral Surgery, Institute of Biomedical Sciences, Nagasaki University, 1-7-1, Sakamoto, Nagasaki, 852-8588 Japan; 3 Basic and Translational Research Center for Hard Tissue Disease, Institute of Biomedical Sciences, Nagasaki University, 1-7-1, Sakamoto, Nagasaki, 852-8588 Japan; 4 Department of Regenerative Medicine Science, Tokai University School of Medicine, 143, Shimokasuya, Isehara, 259-1193 Japan

**Keywords:** Bisphosphonate-related osteonecrosis of the jaw, QQ-controlled PBMNCs, Transplantation, Tooth extraction, Wound healing, Neovascularization, Macrophages

## Abstract

**Background:**

Definitive treatment strategies for bisphosphonate-related osteonecrosis of the jaw (BRONJ) have not been developed. Cell-based therapy is an attractive treatment method for intractable diseases in the medical and dental fields; however, approval has been challenging in dentistry. Recently, we developed quality- and quantity (QQ)-controlled peripheral blood mononuclear cells (PBMNCs) that have anti-inflammatory and pro-angiogenesis effects. The aim of this study was to investigate the effects of QQ-controlled PBMNC transplantation on BRONJ-like lesions in mice.

**Methods:**

To create high-prevalence BRONJ-like lesions, cyclophosphamide (CY) and zoledronate (ZA) were used with tooth extraction. Drug treatment was performed for 5 weeks. QQ-controlled PBMNC transplantation was performed immediately following tooth extraction of both maxillary first molars at 3 weeks after drug administration. Mice were euthanized at 2 weeks post-extraction. Histomorphometric and immunohistochemical analyses, microcomputed tomography assessment, and quantitative polymerase chain reaction evaluation were conducted using maxillae and long bones.

**Results:**

ZA effects on long bones were noted, regardless of CY. Severely inhibited osseous and soft tissue wound healing of tooth extraction sockets was induced by CY/ZA combination therapy, which was diagnosed as BRONJ-like lesions. QQ-controlled PBMNC transplantation reduced BRONJ-like lesions by improving soft tissue healing with increased M1 and M2 macrophages and enhanced neovascularization in the connective tissue of tooth extraction sockets. QQ-controlled PBMNC transplantation also reduced inflammation by decreasing polymorphonuclear cells and *TNF-α* expression in the tooth extraction sockets. Additionally, QQ-controlled PBMNC transplantation partially improved osseous healing of tooth extraction sockets. Interestingly, only 20,000 QQ-controlled PBMNCs per mouse induced these transplantation effects. QQ-controlled PBMNC transplantation did not affect the systemic microenvironment.

**Conclusions:**

Our findings suggest that transplantation of a small amount of QQ-controlled PBMNCs may become novel therapeutic or prevention strategies for BRONJ without any adverse side effects.

## Background

Bisphosphonate-related osteonecrosis of the jaw (BRONJ) is a rare but sometimes severe adverse effect of oral and intravenous bisphosphonates. The incidence of BRONJ following tooth extraction in cancer patients receiving intravenous bisphosphonates is obviously higher when compared with that in osteoporosis patients taking oral bisphosphonates (estimates for the risk of BRONJ; 1.6–14.8% vs. 0.5%, respectively) [1]. A recent systematic review has reported that most BRONJ patients (60.7%) had multiple myeloma or breast cancer [2]. These patients often receive chemotherapy with intravenous bisphosphonate administration that reduces skeletal-related events. Not only bisphosphonate monotherapy, but also chemotherapeutic/bisphosphonate combination therapy, has been reported to be critical risk factors for BRONJ [3]. BRONJ has been reported to worsen oral health-related quality of life [4]. Therefore, the development of treatment and prevention strategies for BRONJ is required. However, useful treatment strategies for BRONJ have not been established due to the difficulty in understanding the exact mechanisms of BRONJ.

Endothelial progenitor cells (EPCs), which are present in the bone marrow and peripheral blood, have been demonstrated to play an essential role in mediating vasculogenic and anti-inflammatory effects in damaged or injured tissues [5]. To efficiently acquire EPCs from peripheral blood, granulocyte- or granulocyte-macrophage colony-stimulating factor (G-CSF and GM-CSF, respectively) is often administered before collection of EPCs, since G-CSF and GM-CSF injections enhance the mobilization of bone marrow EPCs into peripheral blood in animal and clinical studies [6–9]. However, G-CSF and GM-CSF administration induces some adverse effects [10–12]. The selection of EPCs is methodologically complex and highly expensive, as cell sorting using magnetic beads is required in clinical settings [13]. Moreover, it has been shown that the number and regenerative ability of EPCs are reduced with aging and systemic diseases [14, 15]. To overcome these disadvantages, we have developed quality and quantity (QQ)-controlled peripheral blood mononuclear cells (PBMNCs) using serum-free medium containing five growth factors (stem cell factor, thrombopoietin, Flt-3 ligand, vascular endothelial growth factor, and interleukin-6) [16]. Moreover, it has been demonstrated that transplantation of QQ-controlled PBMNCs induces neovascularization and reduces inflammation in mouse ischemic hindlimbs and a bone fracture model in rats [16, 17].

To the best of our knowledge, there are no previous studies investigating the effects of QQ-controlled PBMNC transplantation on BRONJ. We hypothesized that QQ-controlled PBMNC transplantation reduces and/or prevents BRONJ-like lesions in mice based on the rationale of anti-inflammatory and neovascularization effects induced by QQ-controlled PBMNC transplantation. The aim of this study was to investigate the effects of QQ-controlled PBMNC transplantation on tooth extraction socket healing using previously established high-prevalence BRONJ-like lesions in mice [18, 19].

## Methods

### Animal model

**Fig. 1:**
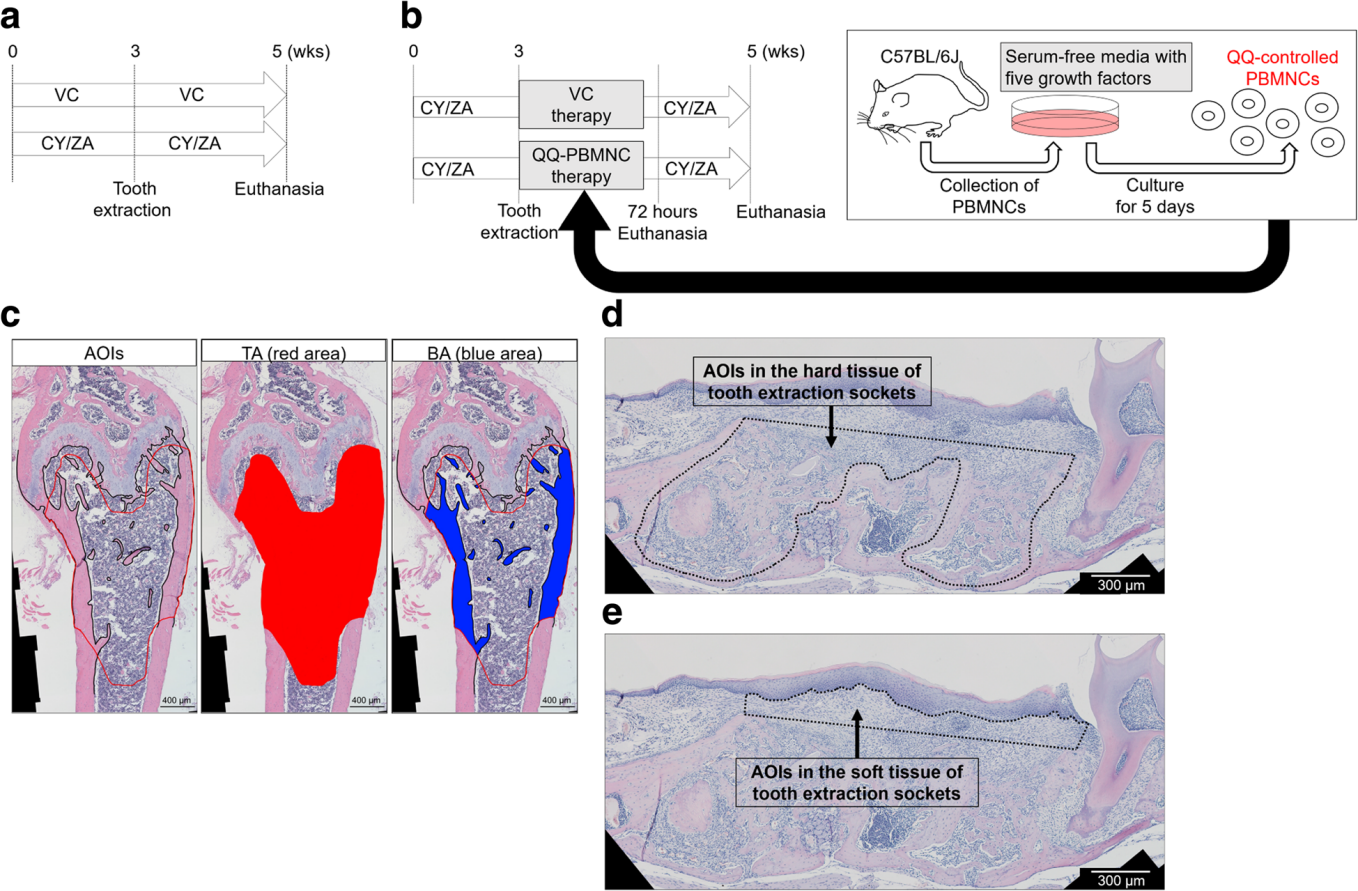
Experimental procedures. **a** Experimental schedule to create high-prevalence BRONJ-like lesions. **b** Experimental schedule for transplantation of QQ-controlled PBMNCs. **c** Area of interests (AOIs) in long bones. AOIs, total area (TA; the red area) and bone area (BA; the blue area). **d** AOIs in the hard tissue of tooth extraction sockets (area surrounded by black dotted line). **e** AOIs in the soft tissue of tooth extraction sockets (area surrounded by black dotted line)

Sixty-two female C57BL/6 J mice (8 to 12 weeks old) were used in this study. A high-prevalence BRONJ animal model was implemented as previously described [18]. Briefly, bisphosphonate (Zometa, designated as ZA; Novartis, Stein, Switzerland) and cyclophosphamide (CY, C3797; Sigma-Aldrich, St. Louis, MO) were administered to mice subcutaneously and intraperitoneally for 5 weeks, respectively. Tooth extraction of maxillary first molars was performed at 3 weeks post-drug administration (ZA, 0.05 mg/kg twice a week; CY, 150 mg/kg twice and once a week before and after tooth extraction, respectively) as follows: The periodontal ligament around the alveolar ridge was disrupted using a dental explorer and a 1/2 Hollenback carver (CVHL1/2 #41 Round, Hu-Friedy Mfg. Co., LLC, Chicago, IL) from both the buccal and palatal sides. Maxillary first molars were very carefully luxated by insertion of the tip of the dental explorer and the Hollenback carver to avoid the mesial root of the first molars with angulation. The luxated first molar was carefully removed using surgical forceps. After tooth extraction, a stereomicroscope was used to confirm whether or not the dental roots of the extracted molar were fractured. Six mice with fractured roots were excluded in this study since the fractured roots would affect wound healing following tooth extraction. Hence, the remaining 56 mice (112 intact tooth extraction sockets were included in this study. Saline was used as the vehicle control (VC). Euthanasia was carried out 2 weeks post-extraction (Fig. 1a; *n* = 7/each group). Animal care and experimental procedures were performed in accordance with the Guidelines for Animal Experimentation of Nagasaki University, with the approval from the Ethics Committee for Animal Research (Approval No. 1708241404-2 and 1610181345-5).

### Isolation, culture, and transplantation of QQ-controlled PBMNCs

Mouse PBMNCs were harvested by cardiac puncture. PBMNC culture was performed with serum-free media containing stem cell factor (Peprotech, Rocky Hill, NJ), thrombopoietin (Peprotech), Flt-3 ligand (Peprotech), vascular endothelial growth factor (Peprotech), and interleukin-6 (Peprotech) for 5 days, according to our previous study [16]. In this study, the cultured cells were defined as QQ-controlled PBMNCs, since the characteristics of these cells have been precisely analyzed and described in our previous study [16]. Transplantation of QQ-controlled PBMNCs (2 × 10^4^ cells/100 μL saline/mouse) was performed via tail vein immediately following tooth extraction in CY/ZA-treated mice (CY/ZA-QQ-PBMNC therapy). The CY/ZA-treated control group received saline administration just after tooth extraction (CY/ZA-VC therapy). Mice were euthanized at 72 h and 2 weeks after transplantation (Fig. 1b; *n* = 7/each group at 72 h post-transplantation, 14/each group at 2 weeks post-transsplantation).

### Microcomputed tomography (microCT) assessment

Left maxillae (*n* = 7/each group) and tibiae (*n* = 7/each group) were dissected, fixed with 10% of neutral formalin solution (MUTO PURE CHEMICALS Co., Ltd., Tokyo, Japan), and scanned by microCT (R_mCT2; Rigaku Co., Tokyo, Japan) using a voxel resolution and an energy level of 20-μm and 90 kV, respectively. Extraction sockets and tibial metaphyseal regions were segmented and reconstructed by semi-manual contouring and were analyzed with TRI/3D-Bon (Ratoc System Engineering, Tokyo, Japan). Trabecular parameters such as bone mass in tooth extraction sockets and long bones, trabecular number (Tb.N), trabecular thickness (Tb.Th), trabecular separation (Tb.Sp), and bone mineral density (BMD) were determined using the direct-measure technique [20, 21].

### Histomorphometric analysis of femora

After fixation of right femora with 10% neutral formalin solution, demineralization was conducted at 4 °C for 21 days using 10% ethylenediaminetetraacetic acid (EDTA; pH = 7.3, FUJIFILM Wako Pure Chemical Co., Osaka, Japan) (*n* = 7/each group). The EDTA solution was exchanged every 3 days. Demineralized femora were paraffin-embedded, and sectioned at a thickness of 5 μm. Hematoxylin and eosin (H-E) staining was performed using the manufacturer’s standard protocol. To observe osteoclasts, tartrate-resistant acid phosphatase (TRAP) staining was conducted (HT15; Sigma-Aldrich). Stained sections were photomicrographed by light microscopy (Axio Scope A1; Zeiss, Carl Zeiss, Gottingen, Germany), and the following parameters were histomorphometrically evaluated using ZEN2 software (Zeiss) and NIH ImageJ (version 1.47; National Institutes of Health, Bethesda, MD): bone area per tissue area [BA/TA (%)] and osteoclast number per linear bone perimeter [N.Oc/BS (#/mm)]. The areas of interest (AOIs) of each parameter ranged from 200 to 2200 μm below the growth plate of the distal femur (Fig. 1c). Three serial sections from each mouse were blindly analyzed by two independent evaluators (SK and KN). All analyzed data were collected and averaged values were used for each mouse.

### Evaluation of gross wound healing of tooth extraction sockets

Occlusal view images of tooth extraction sockets were taken just after euthanasia with Vernier Caliper (Mitutoyo Corporation, Kanagawa, Japan). Borderlines between the epithelium and exposed bone were digitally drawn under high magnification. In this study, the area surrounded by the borderline and the length of the line were defined as wound open area and perimeter, respectively. NIH ImageJ was used for quantitative measurement of the parameters.

### Validation of BRONJ-like lesions by histomorphometry

After fixation of right maxillae, demineralization was performed with 10% EDTA at 4 °C for 21 days. The EDTA solution was exchanged every 3 days. Demineralized maxillae were paraffin-embedded and sectioned at a thickness of 5 μm (*n* = 7/each group). H-E, TRAP, and Masson’s trichrome (386A; Sigma-Aldrich) staining were carried out. AOIs in the tooth extraction sockets were determined as follows: the area surrounded by the line linking the tops of the mesial and distal alveolar ridges, and the curve parallel to the line within 50 μm of the outer line of the laminar dura in the hard tissue of tooth extraction sockets (Fig. 1d). Moreover, the areas of connective tissue, excluding epithelium above the line linking the tops of the mesial and distal alveolar ridges, were also determined as AOIs in the soft tissue of tooth extraction sockets (Fig. 1e). The following parameters were histomorphometrically assessed to validate impaired tooth extraction socket healing showing human-like BRONJ conditions, as previously described [22, 23]: N.Oc/BS (#/mm); osteocyte number in tooth extraction sockets [osteocyte density (#/mm^2^)]; empty osteocyte lacuna number in tooth extraction sockets [empty lacunae (#/mm^2^)]; living bone area, designated as bone with normal osteocytes except for empty and pyknotic osteocyte lacunae [living bone (%)]; necrotic bone in extraction sockets, defined as the portion of bone in which there were greater than or equal to 10 adjacent empty or pyknotic osteocyte lacunae [20, 24] [necrotic bone (%)]; infiltration of polymorphonuclear cells (PMNs) within 100 μm of bone surfaces [PMN infiltration (#/mm^2^)].

### Evaluation of collagen production in the connective tissue of tooth extraction sockets

Demineralized maxillae were paraffin-embedded and sectioned at a 5-μm thickness (*n* = 7/each group). Masson’s trichrome (386A; Sigma-Aldrich) and picrosirius red (Direct red 80; Sigma-Aldrich) staining were performed to evaluate collagen fibers. Stained sections were photomicrographed using light microscopy (Axio Scope A1; Zeiss) for trichrome-stained sections. Type I and III collagen was separately assessed for picrosirius red-stained sections using polarized light microscopy (Axio Lab. A1; Zeiss), as in our previous study [25]. AOIs were defined as the connective tissue area above the line linking the tops of the mesial and distal alveolar ridges. The images were histomorphometrically analyzed with ZEN2 software (Zeiss) and NIH ImageJ: collagen production is defined as collagen fibers in the AOIs [collagen production (%)]; type I collagen is defined as the ratio of yellow-orange area occupying each AOI [type I collagen (%)]; and type III collagen is defined as the ratio of green area occupying each AOI [type III collagen (%)].

### Immunohistomorphometric analyses of oral wound healing

To detect blood vessels and macrophages in the connective tissue of tooth extraction sockets, rat anti-mouse CD31 monoclonal antibody (ab56299; Abcam, Cambridge, MA; 1:100 dilution), rat anti-mouse F4/80 monoclonal antibody (ab16911; Abcam; 1:50 dilution), and rabbit anti-mouse CD206 polyclonal antibody (ab64693; Abcam; 1:50 dilution) were used as primary antibodies. Alexa Fluor 488 goat anti-rabbit IgG and Alexa Fluor 546 goat anti-rat IgG (Invitrogen, Carlsbad, CA; 1:200 dilution) were used as secondary antibodies. Sections were mounted with VECTASHIELD Antifade Mounting Medium with DAPI (H-1200; Vector Laboratories, Burlingame, CA). Stained sections were photomicrographed by fluorescence microscopy (Axio Scope A1; Zeiss) and were histomorphometrically analyzed with ZEN2 software and NIH ImageJ to yield the following parameters: the number of blood vessels [blood vessels (#/mm^2^)]; vessel surface area in the AOIs of soft tissue in the tooth extraction sockets [vessel density (%)] (AOI, 200 × 1000 μm); F4/80^+^CD206^−^ macrophages [F4/80^+^CD206^−^ cells]; and F4/80^+^CD206^+^ macrophages [F4/80^+^CD206^+^ cells] in the AOIs of soft tissue in the extraction sockets (AOI, 200 × 1000 μm).

### Quantitative real-time polymerase chain reaction

First-strand cDNA was synthesized from gingival tissues including tooth extraction sockets on both sides and/or bone marrow from femora at 72 hours and 2 weeks after post-QQ-controlled PBMNC transplantation, as previously described [18] (*n* = 7/each group for both time points). A Thermal Cycler Dice Real Time System (TaKaRa Co., Shiga, Japan) was utilized for quantitative real-time polymerase chain reaction (qPCR) using SYBR green (Invitrogen) with each sample being run in triplicate. Relative gene expression levels of *tumor necrosis factor-α* (*TNF-α*), *interleukin-10* (*IL-10*), *transforming growth factor-β* (*TGF-β*), *interleukin-1β (IL-1β*), *cathepsin K* (*CTSK*) and *TRAP* were normalized against the expression of the housekeeping gene *β-actin* [18] (Table 1).

**Table 1:** Primer sets for qPCR

Primer sets	Forward	Reverse
*β-actin*	TGGGAATGGGTCAGAAGGAC	GGTCTCAAACATGATCTGGG
*TNF-α*	GGCATGGATCTCAAAGACAACC	CAGGTATATGGGCTCATACCAG
*IL-10*	GGTTGCCAAGCCTTATCGGA	ACCTGCTCCACTGCCTTGCT
*TGF-β1*	TTGCTTCAGCTCCACAGAGA	TGGTTGTAGAGGGCAAGGAC
*IL-1β*	TGTGAAATGCCACCTTTTGA	CTGCCTGAAGCTCTTGTTGA
*F4/80*	CAAGACTGACAACCAGACG	ACAGAAGCAGAGATTATGACC
*CTSK*	CAGCAGAACGGAGGCATTGA	CCTTTGCCGTGGCGTTATAC
*TRAP*	CAGCAGCCAAGGAGGACTAC	ACATAGCCCACACCGTTCTC

### ELISA for serum alkaline phosphatase (ALP)

Blood was collected by cardiac puncture immediately following euthanasia. Serum was prepared by centrifugation (2000 rpm × 30 min) and kept at − 80 °C until use. Serum ALP levels were measured using an ALP mouse ELISA kit (BioVision, Milpitas, CA). Samples were run in duplicate (*n* = 5/each group).

### Statistical analyses

The Shapiro-Wilk test was performed to evaluate normality. Student’s *t* test and the Mann-Whitney *U* test were conducted to compare two groups for parametric and non-parametric data, respectively. All statistical analyses were carried out with Systat 13 (Systat Software, Chicago, IL). An α-level of 0.05 was used for statistical significance. All data are represented as means ± SEM.

## Results

### Antiresorptive effects of ZA on long bones in combination with CY

**Fig. 2:**
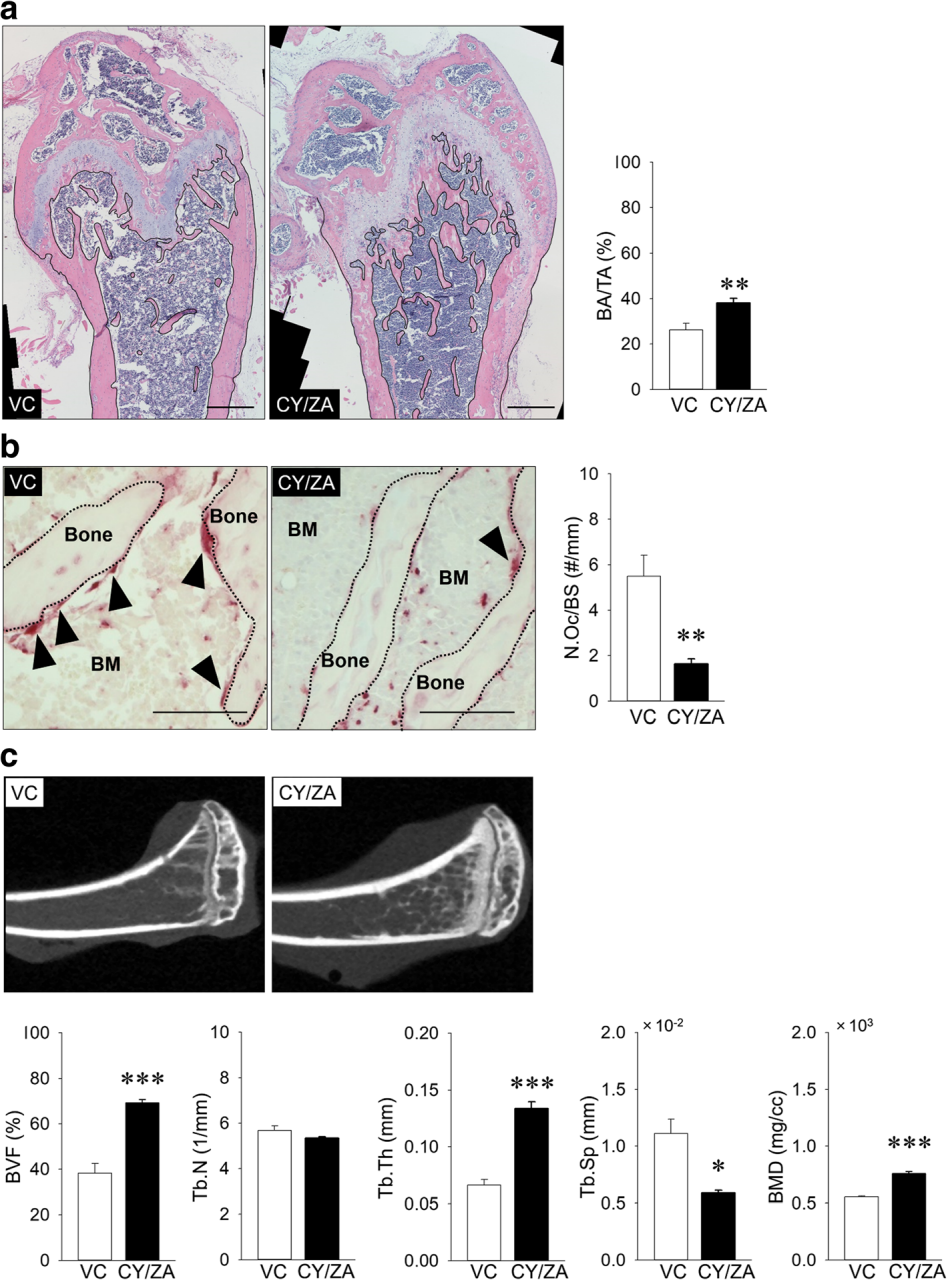
Effects of 5 weeks of cyclophosphamide (CY) and zoledronate (ZA) combination therapy on long bones. **a** Representative H-E-stained images (bar, 400 μm). Bone area per tissue area (BA/TA) was significantly increased in CY/ZA vs. vehicle control (VC) (**p < 0.01). **b** Representative TRAP-stained images (black arrowheads: osteoclasts on bone surface, BM: bone marrow, bar: 100 μm). The number of osteoclasts was significantly decreased in CY/ZA vs. VC (**p < 0.01). **c** Representative microCT images. Bone volume fraction (BVF) was significantly increased in CY/ZA vs. VC (***p < 0.001). Trabecular number (Tb.N) was the same in CY/ZA vs. VC, while trabecular thickness (Tb.Th) was significantly increased in CY/ZA vs. VC (***p < 0.001). Trabecular separation (Tb.Sp) was significantly decreased in CY/ZA vs. VC (*p < 0.05). Bone mineral density (BMD) was significantly increased in CY/ZA vs. VC (***p < 0.001). The Mann-Whitney *U* test was conducted to assess the non-parametric BA/TA data. Student’s *t* test was performed for all other analyzed parameters. *n* = 7/group

First, the effects of CY/ZA combination therapy on long bones were investigated. CY/ZA combination therapy significantly increased bone area when compared with that in VC (Fig. 2a; *p* = 0.009, non-parametric data). This therapy significantly decreased the number of osteoclasts on bone surfaces when compared with that in VC (Fig. 2b; *p* = 0.008). From microCT analysis, CY/ZA combination therapy significantly increased BVF (*p* = 0.000), Tb. Th (*p* = 0.000), and BMD (*p* = 0.000), with a significant decrease in Tb.Sp (*p* = 0.014) when compared with that in VC (Fig. 2c).

### Histopathological findings of tooth extraction sockets in mice treated with CY/ZA

**Fig. 3:**
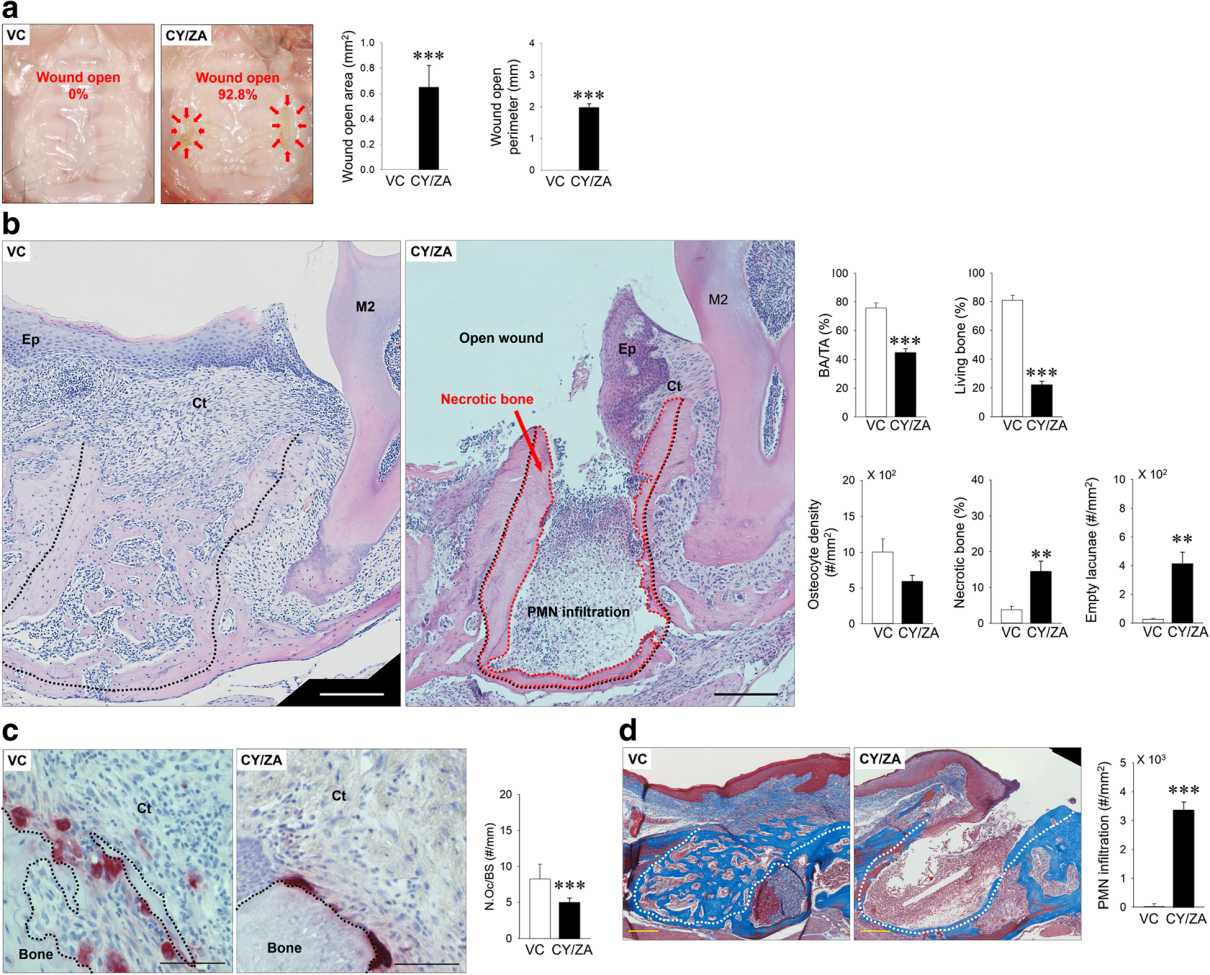
Validation of BRONJ-like lesions induced by CY/ZA combination therapy with tooth extraction. **a** Representative occlusal view of intra-oral photos. Bone exposure without epithelium occurred in 92.8% of tooth extraction sockets (areas surrounded by red arrows). No bone exposure was observed in the VC group. Both open wound area and perimeter were significantly larger in CY/ZA vs. VC (***p < 0.001). **b** Representative H-E stained images of tooth extraction sockets of distal roots (black dotted line: tooth extraction sockets; areas surrounded by red dotted line: necrotic bone with empty lacunae; Ep: epithelium; Ct: connective tissue; M2: second molar; PMN infiltration: polymorphonuclear cell infiltration; bar, 200 μm). BA/TA and living bone area were significantly decreased in CY/ZA vs. VC (***p < 0.001). Necrotic bone and empty lacunae were significantly increased in CY/ZA vs. VC (**p < 0.01). **c** Representative TRAP-stained images (Ct: connective tissue; bar, 100 μm). The number of osteoclasts was significantly decreased in CY/ZA vs. VC (***p < 0.001). **d** Trichrome-stained images of extraction sockets of maxillary mesial roots (white dotted line: extraction sockets of mesial roots; bar, 100 μm). Severe infiltration of polymorphonuclear cells (PMNs) in the tooth extraction sockets was observed in CY/ZA relative to VC (***p < 0.001). Student’s *t*-test was performed for all analyzed parameters. *n* = 7/group

Thirteen out of 14 tooth extraction sockets (92.8%) were not healed without epithelial coverage in CY/ZA, whereas no open wounds were observed in VC. CY/ZA combination therapy significantly increased wound open area and perimeter when compared with those in VC (*p* = 0.000 and 0.000, respectively) (Fig. 3a). CY/ZA combination therapy significantly decreased BA/TA (*p* = 0.000) and living bone (*p* = 0.000), with significant decreases in necrotic bone (*p* = 0.004) and empty lacunae (*p* = 0.001) (Fig. 3b). CY/ZA combination therapy significantly decreased the number of osteoclasts on bone surfaces in the tooth extraction sockets (Fig. 3c; *p* = 0.000). CY/ZA combination therapy significantly increased infiltration of PMNs in the tooth extraction sockets (Fig. 3d; *p* = 0.000).

### The effects of systemic transplantation of QQ-controlled PBMNCs on osseous wound healing of tooth extraction sockets

**Fig. 4:**
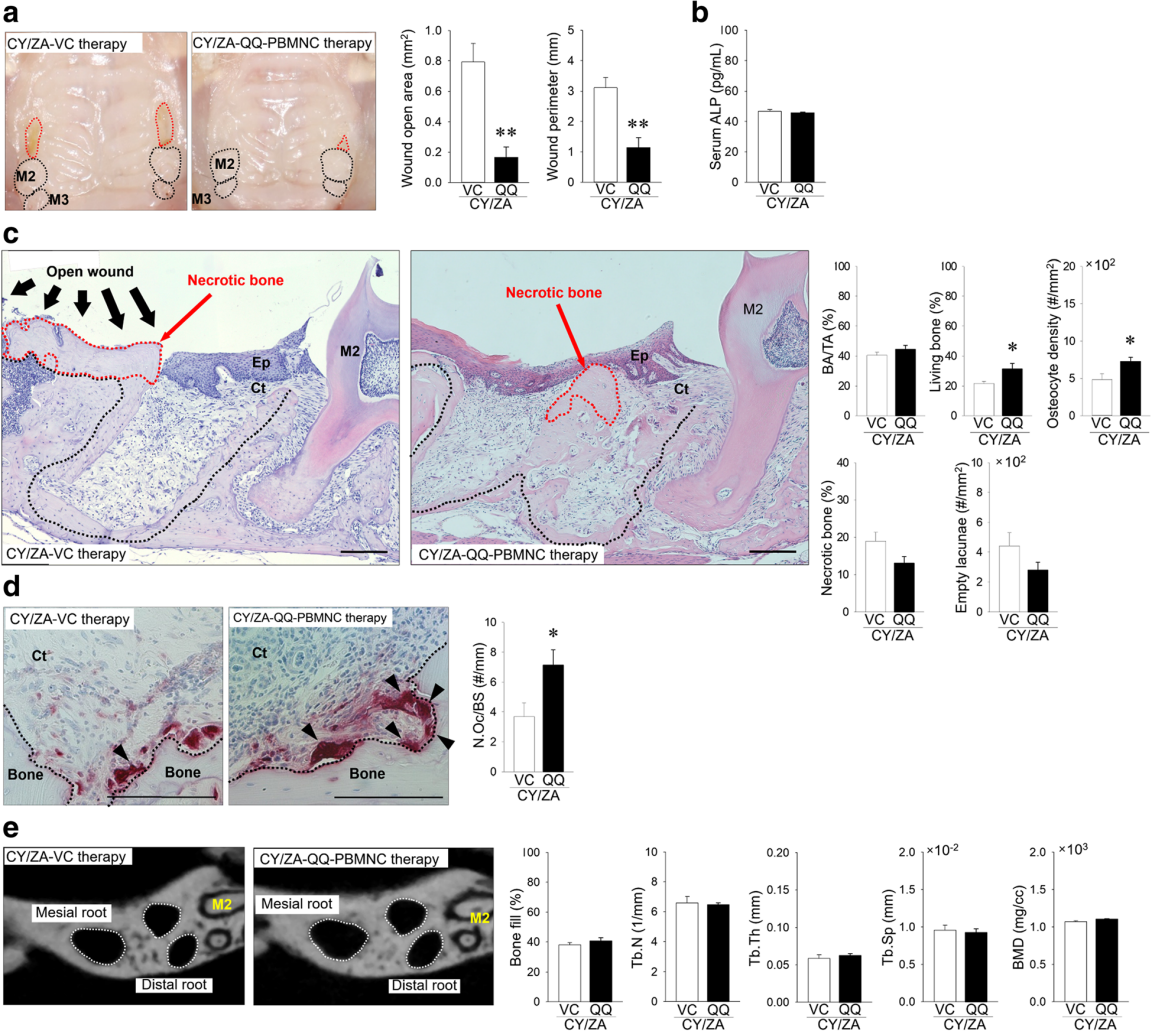
Effects of QQ-controlled PBMNC transplantation on impaired osseous healing at 2 weeks post-extraction. **a** Representative occlusal view of intra-oral photos (red dotted line: open wounds, M2: second molar, M3: third molar). Both wound open area and perimeter were significantly decreased in QQ-controlled PBMNC vs. VC (**p < 0.01). **b** Serum ALP levels were the same between QQ-controlled PBMNC and VC. **c** Representative H-E-stained images of tooth extraction sockets of distal roots (black dotted line: tooth extraction sockets; areas surrounded by red dotted line: necrotic bone with empty lacunae; Ep: epithelium; Ct: connective tissue; M2: second molar; bar, 200 μm). BA/TA was the same between QQ-controlled PBMNC and VC. Living bone area was significantly increased in QQ-controlled PBMNC vs. VC (*p < 0.05). Osteocyte density was significantly increased in QQ-controlled PBMNC vs. VC (*p < 0.05). Necrotic bone area was decreased in QQ-controlled PBMNC vs. VC, however, no statistically significant differences were observed (*p* > 0.05). Empty lacunae were similar between QQ-controlled PBMNC and VC. **d** Representative TRAP-stained images (Ct: connective tissue; black arrowheads: osteoclasts on bone surface; bar, 200 μm). Number of osteoclasts was significantly increased in QQ-controlled PBMNC vs. VC (*p < 0.05). **e** Representative microCT images (white dotted line: mesial and distal roots of tooth extraction sockets; M2: second molar). Bone fill in tooth extraction sockets was the same, regardless of cell transplantation. Trabecular number (Tb.N) was similar between groups. Trabecular thickness (Tb.Th) was the same between groups. Trabecular separation (Tb.Sp) was almost identical, irrespective of QQ-controlled MNC therapy. Bone mineral density (BMD) did not change between groups. Student’s *t* test was performed for all analyzed parameters. *n* = 7/group; QQ: QQ-controlled PBMNC therapy

Next, the effects of systemic QQ-controlled PBMNC transplantation on tooth extraction sockets were investigated. CY/ZA-QQ-controlled PBMNC therapy significantly decreased wound open area and perimeter when compared with those in CY/ZA-VC therapy (*p* = 0.001 and 0.005, respectively) (Fig. 4a). Serum ALP level was the same in CY/ZA-VC therapy and CY/ZA-QQ-controlled PBMNC therapy (*p* = 0.488) (Fig. 4b). CY/ZA-QQ-controlled PBMNC therapy significantly increased living bone and osteocyte density when compared with those in CY/ZA-VC therapy (*p* = 0.036 and 0.03, respectively) (Fig. 4c). CY/ZA-QQ-controlled PBMNC therapy decreased necrotic bone when compared with that in CY/ZA-VC therapy without a statistically significant difference (*p* = 0.082) (Fig. 4c). CY/ZA-QQ-controlled PBMNC therapy significantly restored the number of osteoclasts on bone surfaces in the tooth extraction sockets when compared with that in CY/ZA-VC therapy (Fig. 4d; *p* = 0.048). Bone structural parameters such as bone fill, Tb.N, Tb.Th, Tb.Sp, and BMD were not altered by QQ-controlled PBMNC therapy (Fig. 4e; *p* = 0.456, 0.753, 0.450, 0.731 and 0.731, respectively).

### The effects of systemic transplantation of QQ-controlled PBMNCs on soft tissue wound healing of tooth extraction sockets

**Fig. 5:**
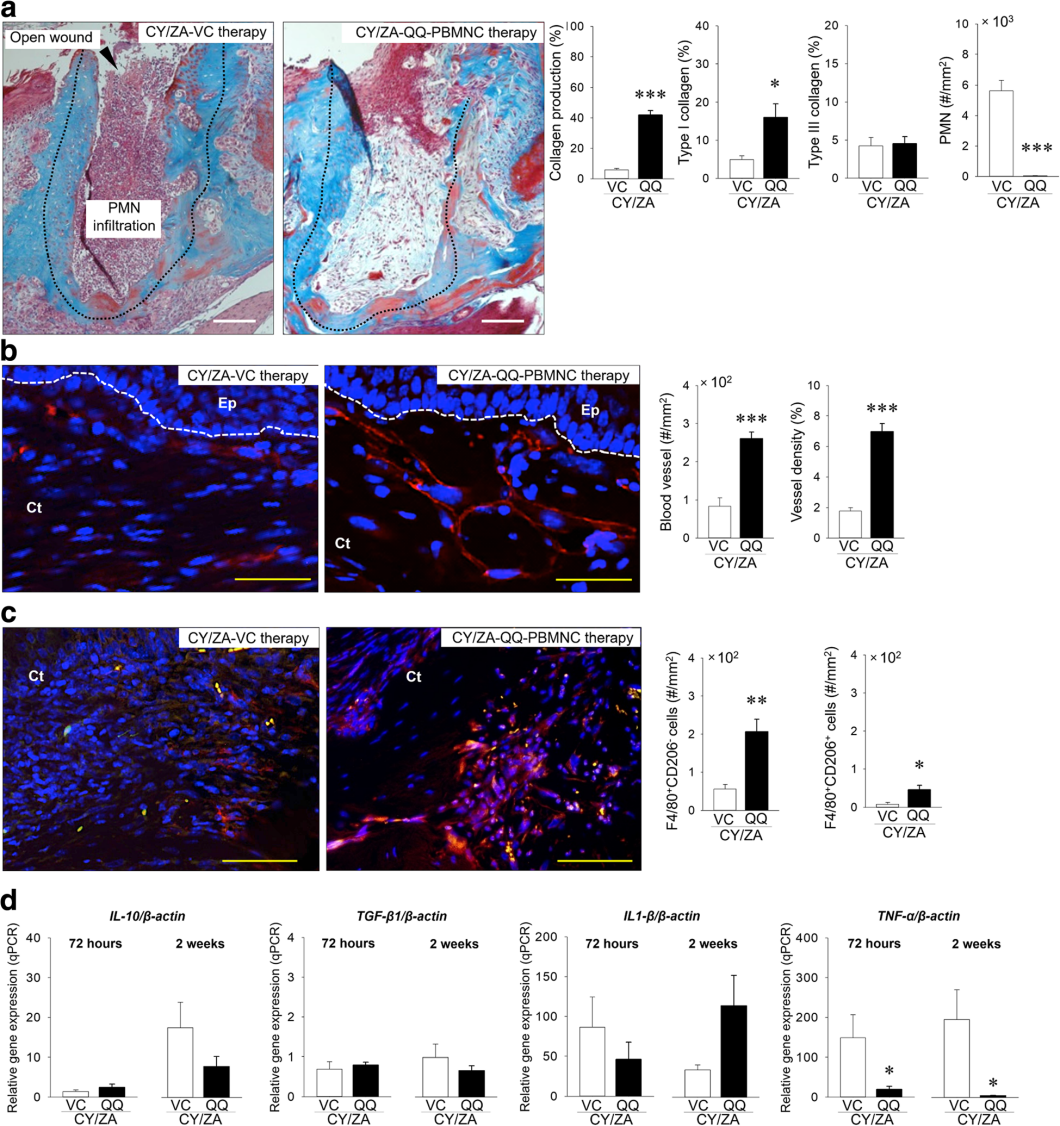
Effects of QQ-controlled PBMNC transplantation on compromised soft tissue healing at 2 weeks post-extraction. **a** Representative trichrome-stained images of tooth extraction sockets of distal roots (black dotted line: tooth extraction sockets; bar, 200 μm). Collagen production in the connective tissue of tooth extraction was significantly increased in QQ-controlled PBMNC vs. VC (***p < 0.001). Notably, production of type I collagen was significantly increased in QQ-controlled PBMNC vs. VC (*p < 0.05), whereas the production of type III collagen was the same between QQ-controlled PBMNC and VC. Polymorphonuclear cells (PMNs) were significantly decreased in QQ-controlled PBMNC vs. VC (***p < 0.001). **b** Representative CD31-immunostained images (red fluorescence: blood vessels; Ep: epithelium; Ct: connective tissue; bar, 50 μm). The number of blood vessels was significantly increased in QQ-controlled PBMNC vs. VC (***p < 0.001). Vessel density was significantly increased in QQ-controlled PBMNC vs. VC (***p < 0.001). **c** Representative F4/80- and CD206-immunostained images (red fluorescence: F4/80^+^ macrophage; orange fluorescence: F4/80^+^CD206^+^ cell; Ct: connective tissue; bar, 100 μm). The numbers of F4/80^+^CD206^−^- and F4/80^+^CD206^+^-cells were significantly increased in QQ-controlled PBMNC vs. VC (***p < 0.01 and *p < 0.05, respectively). **d** Relative gene expression levels of *IL-10*, *TGF-β*, and *IL-1β* were the same between groups at both 72 hours and 2 weeks after cell transplantation. The relative gene expression level of *TNF-α* was significantly decreased in QQ-controlled PBMNC vs. VC at 72 hours and 2 weeks after cell therapy (*p < 0.05). The Mann-Whitney *U* test was used for *IL-1β* analysis. Student’s *t* test was performed for all other analyzed parameters. *n* = 7/group; QQ: QQ-controlled PBMNC therapy

CY/ZA-QQ-controlled PBMNC therapy significantly increased the production of collagen fibers when compared with that in CY/ZA-VC therapy (Fig. 5a; *p* = 0.000). CY/ZA-QQ-controlled PBMNC therapy significantly increased type I collagen, but not type III collagen when compared with those in CY/ZA-QQ-VC therapy (Fig. 5a; *p* = 0.014 and 0.814, respectively). CY/ZA-QQ-controlled PBMNC therapy also significantly decreased PMN infiltration when compared with that in CY/ZA-VC therapy (Fig. 5a; *p* = 0.000). CY/ZA-QQ-controlled PBMNC therapy significantly increased the number of blood vessels and vessel density when compared with those in CY/ZA-VC therapy (Fig. 5b; *p* = 0.000 and 0.000, respectively). Moreover, CY/ZA-QQ-controlled PBMNC therapy significantly increased both the number of M1 and M2 macrophages when compared with CY/ZA-VC therapy (Fig. 5c; *p* = 0.008 and 0.046, respectively). CY/ZA-QQ-controlled PBMNC therapy significantly decreased relative gene expression of *TNF-α* at 72 hours and 2 weeks (*p* = 0.041 and 0.023, respectively) when compared with those in CY/ZA-VC therapy, whereas relative gene expression levels of *IL-10* (72 hours, *p* = 0.308; 2 weeks, *p* = 0.141), *TGF-β1* (72 hours, *p* = 0.647; 2 weeks, *p* = 0.290), and *IL1-β* (72 hours, *p* = 0.386; 2 weeks, *p* = 0.053) did not change at both time points (Fig. 5d).

### Adverse effects of systemic transplantation of QQ-controlled PBMNCs on bone marrow microenvironment

**Fig. 6:**
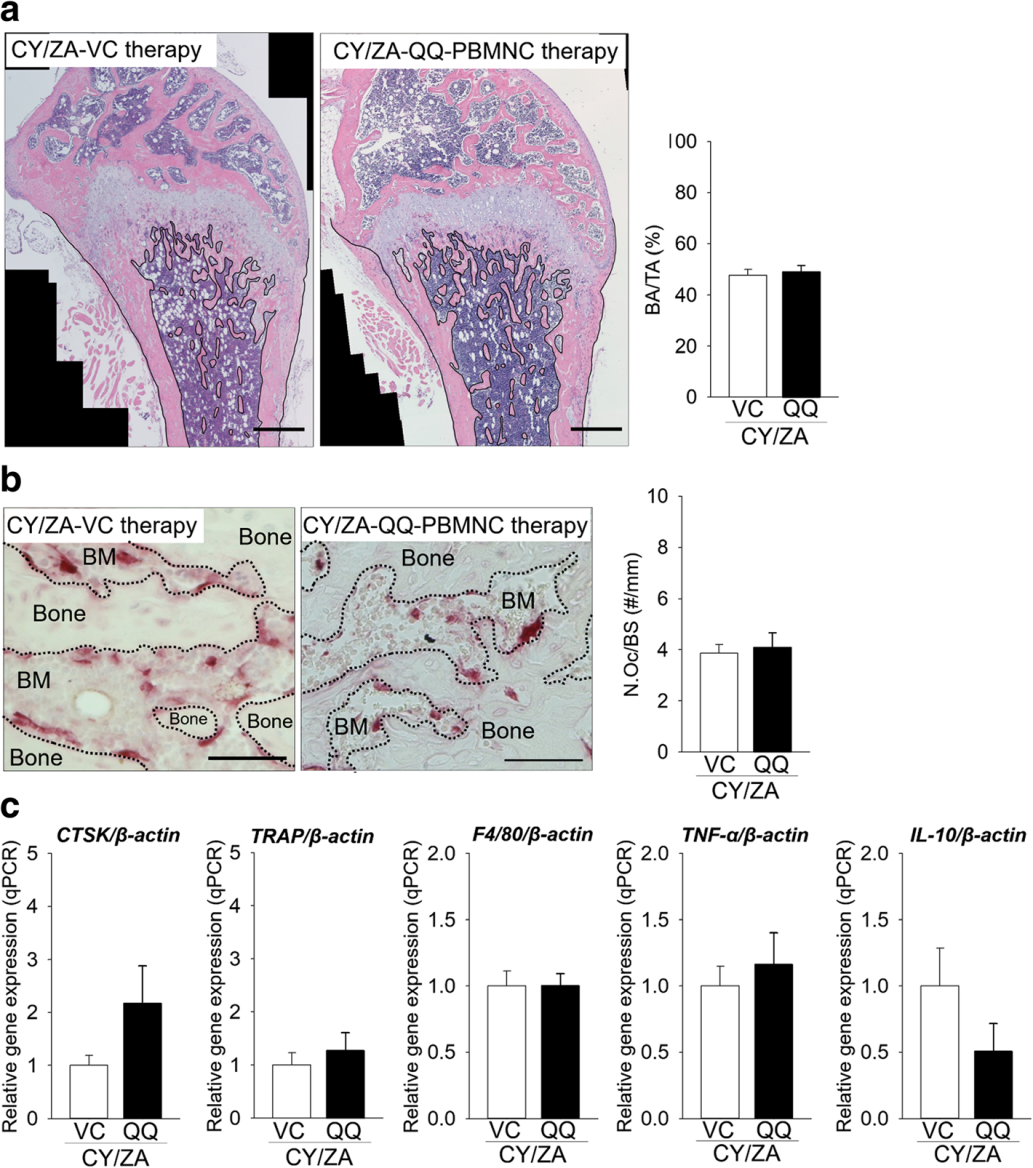
Effects of QQ-controlled PBMNC transplantation on CY/ZA affected-long bones. **a** Representative H-E-stained images (bar, 400 μm). BA/TA was the same, irrespective of QQ-controlled PBMNC transplantation. **b** Representative TRAP-stained images (BM: bone marrow; bar, 100 μm). QQ-controlled PBMNC transplantation did not significantly alter the number of osteoclasts. **c** The relative gene expression levels of *CTSK*, *TRAP*, F4/80, *TNF-α*, and *IL-10* were the same between QQ-controlled PBMNC and VC. The Mann-Whitney *U* test was used for *IL-10* analysis. Student’s *t* test was performed for all other analyzed parameters. *n* = 7/group; QQ: QQ-controlled PBMNC therapy

Finally, adverse effects of systemic transplantation of QQ-controlled PBMNCs on bone marrow were evaluated. CY/ZA-QQ-controlled PBMNC therapy did not affect BA/TA (Fig. 6a; *p* = 0.328) and osteoclast number on bone surfaces (Fig. 6b; *p* = 0.885). Moreover, CY/ZA-QQ-controlled PBMNC therapy did not change relative gene expression levels of *CTSK* (*p* = 0.168), *TRAP* (*p* = 0.549), *F4/80* (*p* = 1.000), *TNF-α* (*p* = 0.179), and *IL-10* (*p* = 0.574) (Fig. 6c)*.*

## Discussion

We demonstrated that systemic transplantation of QQ-controlled PBMNCs reduces BRONJ-like lesions mainly by improving soft tissue wound healing with improved production of type I collagen, enhanced blood vessel formation, suppressed inflammation, and increased M1 and M2 macrophages in the connective tissue of tooth extraction sockets. Moreover, we also showed that QQ-controlled PBMNC transplantation partially restored osseous healing by increasing living bone and decreasing necrotic bone.

The chemotherapeutic drug CY has been used for the treatment of several types of cancers including multiple myeloma in combination with ZA [26]. It has been reported that CY induces osteoporosis while disturbing both osteoblastogenesis and osteoclastogenesis [27]. In this study, the effect of ZA on osteoclasts in long bones exceeded that of CY, indicating that ZA had a pharmacological effect on bone even when CY was systemically administered in C57BL/6 J mice.

Impaired tooth extraction socket healing is diagnosed as BRONJ in patients taking bisphosphonates when open wounds persist for more than 8 weeks in humans [3]. In this study, sustained open wounds for 2 weeks were designated as BRONJ-like lesions, since osseous and soft tissue wound healing of tooth extraction sockets in mice is faster than in humans [28, 29]. Moreover, open wounds have been demonstrated to be sustained for 4 weeks post-extraction in this animal model [18, 19]. In mice, important events in the connective tissues of tooth extraction sockets occur within 2 weeks during the wound healing process [28]. Thus, in the present study, the effect of QQ-controlled PBMNC transplantation on tooth extraction socket healing was evaluated at 2 weeks post-extraction. The validation of BRONJ-like lesions in mice was conducted according to the validation of BRONJ in humans [3, 22, 23, 30] and our previous studies [18–20]. A systematic review has reported that BRONJ frequently occurs in multiple myeloma patients [2]. Chemotherapeutic drugs such as CY are used to treat multiple myeloma, although other therapeutic protocols are utilized for this disease [31]. Therefore, from a clinical viewpoint, the animal model used herein is thought to be appropriate for evaluating transplantation effects of QQ-controlled PBMNCs on tooth extraction socket healing in mice.

QQ-controlled PBMNC transplantation significantly decreased wound open area and perimeter of tooth extraction sockets with alterations in bone parameters obtained from histomorphometric analysis, but not microCT analysis. It has been reported that QQ-controlled PBMNC transplantation promoted osseous healing with enhanced angiogenesis in long bone fracture sites [17]. In this study, amelioration of BRONJ-like lesions was not observed following systemic administration of PBMNCs (data not shown). Moreover, PBMNC transplantation did not produce neovasculogenic effects on soft tissue wounds (data not shown). Hence, accelerated vasculogenesis by QQ-controlled PBMNC transplantation may be associated with the partial enhancement of bone healing of tooth extraction sockets. Additionally, QQ-controlled PBMNC transplantation significantly recovered osteoclast numbers. Recovery of osteoclasts may also contribute to partial bone healing. However, serum ALP activity, which is an indirect measure of bone turnover, was not altered. More than 2 weeks may be required for extraction sockets to be fully filled with newly formed bone following QQ-controlled PBMNC transplantation and would involve the activation of bone turnover coupled with osteoblasts and osteoclasts. In this study, only 20,000 QQ-controlled PBMNCs were used for transplantation, although the previous studies used a local injection of 2 × 10^5^ QQ-controlled PBMNCs rather than systemic injections [16, 17]. The transplantation route and/or number of QQ-controlled PBMNCs may be correlated with partial bone filling of tooth extraction sockets in this study.

Macrophages are recruited into wounds after open wounds are created by injuries. Macrophages phagocytose debris and bacteria, resulting in wound cleaning and preparing antigen presentation for subsequent immune responses [32]. Recent reports have indicated that M1 macrophages are required to induce inflammatory reactions during the early phases of the wound healing processes, whereas M2 macrophages are needed to repair wounds during the late phases [33]. Macrophages also secrete pro- and anti-inflammatory cytokines, several types of growth factors and cytokines during the wound healing processes; however, M1 and M2 macrophages produce different types of healing-related proteins [33]. On the other hand, the formation of new blood vessels is an absolute requirement for wound healing. Blood vessels supply nutrition and oxygen, which are requisite for wound repair [34]. In the present study, QQ-controlled PBMNC transplantation contributed to the promotion of soft tissue healing around tooth extraction sockets with increased blood vessels, enhanced numbers of M2 (F4/80^+^CD206^+^ cells) and M1 (F4/80^+^CD206^−^ cells) macrophages, increased collagen production, and decreased PMN infiltration. Suppressed relative expression of *TNF-α* in soft tissue wounds at 72 hours and 2 weeks after cell transplantation suggests that the effectiveness of QQ-controlled PBMNC transplantation on soft tissue healing begins at the early phase of the wound healing processes; however, other pro- and anti-inflammatory-related genes did not change at either time point.

Our previous study demonstrated that systemic transplantation of non-cultured stromal vascular fraction (SVF) cells also improved tooth extraction socket healing by increasing the ratio of M2/M1 macrophages and upregulating angiogenesis in developed BRONJ-like lesions in mice [18]. It has been shown that EPC transplantation ameliorated excisional back-wounds by increasing macrophage numbers and neovascularization [35]. It has also been shown that transplantation of bone marrow-derived mesenchymal stem cells (BMMSCs) rescued BRONJ-like lesions in mice [36]. These findings are in accordance with our present findings. However, the number of transplanted EPCs (1 × 10^5^ cells per wound) [35] and QQ-controlled PBMNCs (2 × 10^4^ cells/mouse) used in this study was less than 1/50 to 1/400 when compared with that of transplanted BMMSCs (1 × 10^6^ cells/mouse) [36] and SVF cells (4 × 10^6^ cells/mouse) [18]. Moreover, it has been demonstrated that the effect of QQ-controlled PBMNC transplantation on mouse ischemic diseases was superior to that of EPC transplantation [16]. Therefore, transplantation of a small number of QQ-controlled PBMNCs, but not EPCs, could produce a higher potential for macrophage migration and new vascularization in injured tissues when compared with a large amount of SVF cell- and BMMSC-transplantation. These advantages of QQ-controlled PBMNC transplantation may resolve some problems associated with cell therapies such as cost-effectiveness, safety, culture duration, ethics and adverse effects before and after transplantation.

Transplanted QQ-controlled PBMNCs have been demonstrated to play various roles in wound healing since they contain several distinct cell populations, such as EPCs, T-lymphoid cells (i.e., Th2 cells and regulatory T cells), stem cells, and M2 macrophages [16]. Our present study showed that transplanted QQ-controlled PBMNCs contribute to the recruitment of macrophages and increase angiogenesis in the connective tissue of tooth extraction sockets. Transplanted QQ-controlled PBMNCs may activate host stem cells and/or progenitor cells, resulting in the promotion of cell differentiation into cell types such as macrophages and vascular endothelial cells. QQ-controlled PBMNCs may also differentiate into M1 and/or M2 macrophages in the connective tissues of tooth extraction sockets.

The absence of adverse side effects is one of the most important factors in performing cell-based therapy for intractable diseases. In this study, systemic transplantation of QQ-controlled PBMNCs did not produce adverse effects on the bone marrow microenvironment, which suggests that QQ-controlled PBMNCs could be safely and systemically transplanted into BRONJ patients.

## Conclusions

In summary, acknowledging the limitations of this study due to unknown characteristics of transplanted QQ-controlled PBMNCs, QQ-controlled PBMNC transplantation represents a new treatment strategy for BRONJ that warrants further development of its implementation. In this study, we demonstrated that BRONJ-like lesions were reduced by systemic transplantation of QQ-controlled PBMNCs with the promotion of hard and soft tissue wound healing. We also showed that QQ-controlled PBMNC transplantation recruited M1 and M2 macrophages into the connective tissue of tooth extraction sockets and accelerated neovascularization of wounds, which may contribute to promoted wound repair.

## Data Availability

All data generated or analyzed during the current study are included in this published article.

## Abbreviations

ALP: Alkaline phosphatase
AOI: Area of interest
BA/TA: Bone area per tissue area
BMD: Bone mineral density
BMMSC: Bone marrow-derived mesenchymal stem cell
BRONJ: Bisphosphonate-related osteonecrosis of the jaw
*CTSK*: Cathepsin K
CY: Cyclophosphamide
ELISA: Enzyme-linked immunosorbent assay
EPC: Endothelial progenitor cell
G-CSF: Granulocyte-colony stimulating factor
GM-CSF: Granulocyte-macrophage colony-stimulating factor
H-E: Hematoxylin and eosin
*IL-10*: Interleukin-10
*IL-1β*: Interleukin-1β
microCT: Microcomputed tomography
N.Oc/BS: Osteoclast number per linear bone perimeter
PBMNC: Peripheral blood mononuclear cell
PMN: Polymorphonuclear cell
qPCR: Quantitative real-time polymerase chain reaction
QQ-controlled PBMNC: Quality- and quantity-controlled peripheral blood mononuclear cell
SVF: Stromal vascular fraction
Tb.N: Trabecular number
Tb.Sp: Trabecular separation
Tb.Th: Trabecular thickness
*TGF-β*: Transforming growth factor-β
*TNF-α*: Tumor necrosis factor-α
TRAP: Tartrate-resistant acid phosphatase
ZA: Zoledronate
